# Manifestations and drivers of mistreatment of women during childbirth in Kenya: implications for measurement and developing interventions

**DOI:** 10.1186/s12884-017-1288-6

**Published:** 2017-03-28

**Authors:** Charlotte E. Warren, Rebecca Njue, Charity Ndwiga, Timothy Abuya

**Affiliations:** 1 Population Council, 4301 Connecticut Avenue NW Suite 280, Washington DC, USA; 2 Population Council, PO Box 17643 - 00500, Nairobi, Kenya

**Keywords:** Disrespect and abuse, Mistreatment, Childbirth, Kenya

## Abstract

**Background:**

Disrespect and abuse or mistreatment of women by health care providers in maternity settings has been identified as a key deterrent to women seeking delivery care. Mistreatment includes physical and verbal abuse, stigma and discrimination, a poor relationship between women and providers and policy and health systems challenges. This paper uses qualitative data to describe mistreatment of women in Kenya.

**Methods:**

Data are drawn from implementation research conducted in 13 facilities and communities. Researchers conducted a range of in-depth interviews with women (n-50) who had given birth in a facility policy makers health managers and providers (n-63); and focus group discussions (19) with women and men living around study facilities. Data were captured on paper and audio tapes, transcribed and translated and exported into Nvivo for analysis. Subsequently we applied a typology of mistreatment which includes first order descriptive themes, second and third-order analytical themes. Final analysis was organized around description of the nature, manifestations and experiences, and factors contributing to mistreatment.

**Results:**

Women describe: their negative experiences of childbirth; frustration with lack of confidentiality and autonomy; abandonment by the providers, and dirty maternity units. Providers admit to challenges but describe reasons for apparent abuse (slapped on thighs to encourage women to focus on birthing process) and ‘detention’ is because relatives have abandoned them. Men try to overcome challenges by paying providers to ensure they look after their wives. Drivers of mistreatment are perpetuated by social and gender norms at family and community levels. At facility level, poor managerial oversight, provider demotivation, and lack of equipment and supplies, contribute to a poor experience of care. Weak or non-existent legal redress perpetuate the problem.

**Conclusion:**

This paper builds on the expanding literature on mistreatment during labour and childbirth –outlining drivers from an individual, family, community, facility and policy level. New frameworks to group the manifestations into themes or components makes it increasingly more focused on specific interventions to promote respectful maternity care. The Kenya findings resonate with budding literature – demonstrating that this is indeed a global issue that needs a global solution.

## Background

The quality of the relationship between pregnant women and health providers is a critical factor that affects the use of skilled care at birth. Disrespect and abuse (D&A) of pregnant women by healthcare and other staff in maternity setting [1–5] has been identified as a key deterrent to women seeking facility-based deliveries [1, 3, 6–9] and associated with lower satisfaction with quality of delivery services [1]. Bowser and Hill described seven manifestations of D&A; physical abuse, non-confidential care, non-consented care non-dignified care, discrimination, abandonment, and detainment in facilities, drawn from the literature available in 2010 [3]. Since then developments to further define and measure the prevalence of D&A, and to test interventions to minimize its effect indicate the importance of the issue globally [1, 5–7, 10–12]. The first studies measuring the prevalence of D&A describe anything from 20 to 78% of women reporting some kind of mistreatment (depending on context and measurement methodology) [4–6, 10].

Recently, Bohren et al released a systematic synthesis of 65 studies from 34 countries whereby findings were organized under seven domains: physical abuse, sexual abuse, verbal abuse, stigma and discrimination, failure to meet professional standards of care, poor rapport between women and health providers, and health system conditions and constraints [1]. This paper uses these typologies to describe the types of mistreatment experienced by women during childbirth, and the perceived drivers of this mistreatment in Kenya.

## Methods

Data are drawn from the *Heshima* project, an evidence-based participatory implementation research study, conducted in 13 facilities and surrounding communities in Kenya [13]. The 13 purposefully selected facilities constituted different facility types (public, private, faith-based) and levels of care, comprised of three referral hospitals, three district hospitals, two faith-based hospitals, two maternity homes, and one health center. Four facilities were rural and nine were in urban or peri-urban areas. At the time of data collection women were charged for maternity services.^1^ The number of deliveries per month ranged from 36 in the health center to over 1000 in the largest referral hospital. A detailed description of the methodology used for baseline and outcome data collection (both qualitative and quantitative), is described elsewhere but include measuring the prevalence of D&A through exit interviews, provider interviews and client-provider observations [5, 13].

Qualitative data were collected between August and October 2011 to both understand the taxonomy around D&A and to inform the development of interventions. Researchers conducted 19 focus group discussions (FGDs) with women, men, civil society and community leaders recruited from the catchment areas of twelve facilities (selected from four counties) and one maternity hospital in Nairobi. Participants included women who had given birth (either at home or in a health facility), family members, and local women’s and civil rights groups. Potential participants were recruited from the communities around the health facilities by a research assistant in conjunction with community leaders and invited to participate in a focus group discussion held at a nearby location. A same sex moderator conducted the session, accompanied by a note-taker, who was also responsible for administering the tape recorder. Participants were informed that the session would be taped, and that no personal identifiers will be used during discussions. The focus of discussion was: general problems faced by pregnant women, motivations for giving birth in health facility and selection/use of the particular facility; attitudes towards health facility deliveries; quality and satisfaction with communication/interaction with the provider; perceptions of health system stigma and discrimination towards those accessing maternal health services. Sixty-one in-depth interviews (IDIs) were conducted with policy makers, health managers, health providers, community health workers and traditional birth attendants (TBAs). In-depth interviews with women were also conducted with 17 women who reported D&A during the exit survey and 38 who did not report any D&A at baseline. Table 1 summarizes the data sources and study participants. Given that the focus of the research was to find out the types of D&A to develop interventions to mitigate mistreatment, we only describe the results from those who experienced D&A. The objectives of the interviews was to collect information similar to that collected through FGDs, with additional information on system level and governance factors that may contribute to D&A [13].

**Table 1:** Data sources and study participants

Data Sources	Study groups	Number
In-Depth Interviews (IDIs)	Policy makers: national health program managers, professional associations (nursing, medical)	23
	Health program managers at county and sub county level; maternity unit in-charges (nurses, midwives and doctors)	20
	Health providers (nurses, midwives, doctors)	11
	Community health workers and traditional birth attendants	9
	Women who delivered at a facility in the last 6 months who experienced D&A (17). Women who delivered at a facility in the last 6 months who experienced D&A (33).	50
Focus Group Discussions (FGDs)	Single and multi-parity women (separate groups)	9
	Men in the community	5
	Community members/Opinion leaders (chiefs, elders)	5

### Ethical review

All data collection instruments and procedures were pilot-tested among groups with similar characteristics to the study populations and modified accordingly. The study tools were also translated and pretested in the relevant local language. A structured consent form administered in participants’ preferred language, conveyed the objectives, procedures, benefits, and risks of the study. Written consent was obtained from all participants. None of the participants were minors. The research protocol was reviewed by key stakeholders and ethical clearance was granted by the Kenya Medical Research Institute (KEMRI) Ethical Review Board (approval SCC No: 288), the Population Council’s Institutional Review Board (No: 517), and the Division of Reproductive Health, Ministry of Health, Kenya.

#### Analysis

Qualitative data was captured on paper and audio tapes, translated and transcribed into MS Word, before being exported into QSR NVivo 10 software for management and analysis. Data analysis was conducted by two researchers. A thematic framework approach was adopted to classify and organize data into key themes, concepts and emergent categories. The initial codebook was constructed through inductively derived themes (e.g. neglect and abandonment, bribery) and deductively on the Bowser and Hill conceptual model, which we contextually explored and adapted to the Kenyan setting. This paper further applies the typology of mistreatment of women during childbirth as described by Bohren et al which includes first-order descriptive themes, second- and third-order analytic themes [2]. The first order themes describe specific events or instances of mistreatment. The second and third order analytical themes further classify these first order themes into meaningful groups based on their common attributes. The third-order themes are ordered from the level of interpersonal relations through the level of the health system [2].

We triangulated qualitative data through comparisons of analysis charts within and across sites to look for similarities and differences to support identification of key issues around abuse and disrespect. Final qualitative analysis was organized around a description of the nature, manifestations and experiences at baseline and factors contributing to any mistreatment.

## Results

In response to a generic question at the beginning of the FGDs and IDIs on the problems women face in accessing maternal health services themes that emerged included socioeconomic barriers: poverty, distance to health facilities, lack of and cost of transport – especially at night – and the cost of delivery services. However, the results reported here are around mistreatment within health facilities.

The results are presented in two sections. The first describes mistreatment themes that emerged from the data, grouped by the third order themes outlined by Bohren et al which are 1) physical abuse, 2) verbal abuse, 3) stigma and discrimination, 4) failure to meet professional standards of care, 5) poor rapport between women and health providers and 6) health system conditions and constraints [2]. There were no reports of sexual abuse in the data. Table 2 summarizes the types of mistreatment by order of themes. The second section describes the perceived drivers of the manifestations of the mistreatment.

**Table 2:** Manifestations of mistreatment of women during childbirth in Kenya

Third order themes	Second order themes	Illustrative manifestations of experiences and reports of first order themes from Kenya		
		Women	Men	Health providers (HPs)/managers
Intentional mistreatment: use of violence, physical, verbal, negligent withholding of care				
Physical abuse	Use of force	Slapping/pinching	Beaten by HPs	Slap to save a woman’s life
	Physical restraint	Pushing my thighs	Helped to make the woman cooperate or obey	To make room for baby to come out if mother is closing legs. Fear of being reprimand for poor out pregnancy comes
Sexual abuse	Sexual abuse	Not recorded in any data from this study		
Verbal abuse	Harsh language Threats and blaming	Insulting language; Threatening and insulting relatives/caretaker Women blamed for negative outcomes Reprimanding client if she calls for help	Insults from health providers to women and caregivers	Harsh words ‘helps’ women and relatives cooperate; You must appear tough to gain cooperation Personal attitude
Stigma and discrimination	Discrimination based on ethnicity, socioeconomic status	Women ‘blamed’ for high parity, age and socioeconomic status Tribalism/ethnicity	Devalues my partner/wife and I or my community	Women take too long to understand. HPs overworked, stereo typing, negative attitude and values ‘*that tribe behavior/react like that*”
		HIV positive women avoided or abandoned	Men forced to take HIV test	Fear, stigma lack of knowledge
Failure to meet professional standards of care	Lack of informed consent for physical exam and procedures	HPs discuss the examination results with others	Devalues my wife and I	Rooms do not offer audio privacy
		Student allowed to do episiotomy ‘badly’ Frequent vaginal examinations/multiple tests by HPs and students		Student must learn on clients: too many students must achieve skills in a short time. Lack of HPs skills and confidence.
	Lack of confidentiality and privacy	Examination, delivery and treatment required to undress without curtains or partitions	Bed sharing	Lack of curtains Too many clients Limited space
		Women have to give personal information in public (within hearing distance of others)		Over crowding
	Neglect and abandonment	Older/higher parity women report left to deliver on their own as HPs abandon them due to their previous birth experience	HPs refuse to help women in labor if not come with drugs, supplies, money. Men rush to buy them.	Too busy, overworked, uncooperative mother. Poor staff attitude, “*have done my shift for the day*”, lack of team work. Inadequate supervision, Demotivated.
		Ignoring clients’ calls for help	HPs not available or at night	
		Doctor not available to conduct cesarean section	No doctor available	Absenteeism; report late on duty, no housing for doctors; lack of transport.
		HPs not responding to client when in pain	Some women too afraid of pain	Poor staff attitude; Lack of professional ethics; Poor leadership
Poor rapport between women and health providers	Lack of autonomy	Not involved in decision making in my care Used language that I could not understand Lack of food/drink. No bathing facilities		Poor staff attitude. Too busy to explain, they do not get even if you explained, Ineffective counseling/communication.
	Detainment	Lack of money makes mothers avoid going to hospital	Women are detained	Clients are abandoned by their relatives in hospital.
	Ineffective communication	Not given information about my care Do not understand the need for frequent vaginal examinations	Not consulted or informed about my wife’s progress or babies condition	HPs have no time to discuss procedures due to high workload Clients do not understand
Structural disrespect deviations				
Health system conditions an constraints	Lack of resources	Lack of equipment. bed sharing, Lack of curtains/clean linen	Facility request money to buy drugs	Inadequate supplies, lack of funds, misuse of funds, lack of maintenance, poor planning and forecasting
		No water for bathing, dirty bathrooms No food		
		No support staff Staff shortage/no supervision/poor leadership. Made to clean up	Have to buy the drugs and supplies	Shortages Staff and equipment and supplies
	Facility culture	Too few staff	Staff not supervised	Staff shortages Ineffective supervision
	Corruption/bribery	You must stretch your hand Some behave in a way that they want to be bribed	Pay bribe to get own bed	Poor and delayed pay. Accepted norm- everyone is doing it anyway

The illustrative manifestations (first-order themes describing specific events or instances of mistreatment) presented in this table are drawn from the study findings in Kenya. We have adapted these to the framework developed by Bohren et al [2] using a global evidence-based typology (third, second and first order themes) of mistreatment of women during childbirth. The second- and third-order themes classify first-order themes into meaningful groups based on common attributes. The third-order themes are ordered from the level of interpersonal relations through the level of the health system

*HP* health provider

### Types of disrespect and abuse or mistreatment experienced by women during childbirth

#### Physical abuse

Majority of men and women identified slapping, beating, ‘tossing around’ and pushing as examples of physical abuse, for example: *“She slapped me on the thighs and being tossed around in unfriendly manner but we find it normal to us” (FGD women).* However, the meaning attached to the act during childbirth varied. For example, some of the younger women felt that slapping by health providers is sometimes justified as it is done to ensure the women cooperate and focus on the birth process. This view is also held by health providers who reported that they did not perceive slapping clients as dehumanizing, but considered it an act to help the woman save her life and that of the baby:

*“The issue of mothers saying nurses are bad must be dealt with in the community. They should be informed that nurses are not bad. Ideally any mother who has delivered in a hospital is slapped on the thighs to facilitate or encourage her to push because if the mothers do not push the danger is obvious” (IDI, Service provider).*

Although physical abuse appears to be normalized by both women and health providers, it does deter women from seeking a facility delivery as was illustrated by men and health providers:

*“For me, my opinion is that you may take your wife to hospital the first time but she is afraid to go back because they are usually beaten by the sisters. Once that happens the second time she refuses to go to hospital, she decides to give birth at home” (FGD, Men).*

*“…Some tell of sad stories about their deliveries and they even think that they should not give birth anymore because of the abuse and mistreatment that they receive. A friend of mine was taken to the district hospital but her labor was prolonged which was frustrating the nurses. This made them impatient and abusive to her by slapping and beating her” (IDI, Service provider).*

#### Verbal abuse

Health providers’ use of harsh language, both insulting and shouting at women and their caregivers was a common behavior described by women, men and TBAs. Others reported that health providers blame women for negative outcomes, and reprimand women if they call for help:

*“I was young when I went to deliver my first baby. Instead of being assisted, the nurses kept insulting me (you enjoyed doing it, why are you screaming now), don’t try and scream here. I can never go back to facility XX. The nurses are just there not helping; you wonder if it’s a hospital you were brought to?” (FGD, Women).*

*“It depends on the hospital, like in XX my wife delivered our first child there and she said that she will never go back there… She was asked why she had decided to give birth while still young, so she was really insulted… she said that she will never go back there since those nurses do not have public relation of even telling her what to do and yet this was her first child… she said those nurses do not have customer care” (FGD, Men).*

*“You know pregnant women when they are talked to in a bad way they don’t forget” (IDI, traditional birth attendant).*

Some health providers ‘excused’ this behavior by reporting that it is necessary to be harsh and rude in order to ensure cooperation.

*“Harsh words ‘help’ women and relatives cooperate” (IDI Provider).*

#### Stigma and discrimination

There were two types of discrimination that emerged from the data—discrimination based on socio-demographic status and medical conditions.

##### Discrimination based on socio-demographic characteristics

The types of discrimination were based on parity, socioeconomic status and age. Multiparous women reported that they were mostly abandoned to deliver by themselves as they ‘knew what to do’ having delivered before. Women from poor neighborhoods are labeled ‘poor’ by the health providers who then withhold information regarding procedures and treatment. Many respondents reported that adolescent mothers frequently suffered verbal abuse. Some health providers believe that teenage mothers have defied the community norms by conceiving before the ‘appropriate community age’ and it is somehow justified to be harsh with them. Moreover, the ethnic background of the provider and the clients can dictate their interaction; if they belong to the same ethnic group they get better treatment and are not abandoned or required to bribe.

*“… They, [health providers] say ‘she is a XX tribe leave her she is stupid even if she is in pain’. Until a lady of community XX screams then they can listen to her” (FGD, Women).*

*“Just to add, another thing that can bring problems is tribalism in hospitals. If you are not of her tribe then she will treat you badly, so this also contributes to the poor relationship with the nurses” (FGD, Women).*

Some clients do not seek care in a facility if the health providers are known to be a different ethnicity – forcing the client to travel past the nearest facility. This was more common in the areas worst hit following the post-election violence in 2008. Women in these areas reported that they prefer to visit facilities that are outside their home areas to ensure they get health providers from the same ethnic group or they delivered at home or with a traditional birth attendant.

##### Discrimination based on medical conditions

There were reports of women refusing to attend antenatal care (ANC) services for fear of being tested for HIV and their status made known to people from their community. FGDs with men, women and civil society groups also reported that people in general fear that others might get to know their HIV status if positive because most health providers are careless and often disclose such information.

*“This woman was HIV positive; she was taken to XXX hospital by community health workers from this village, then it was at night….that woman was neglected. They said the woman was neglected, she was almost due but was told to sit on the bench and wait. …the mistake maybe, the woman wasn’t attending antenatal clinic…. so it wasn’t easy*.” *(IDI Community health worker).*

#### Failure to meet professional standards of care

Three main themes (second order) were identified here: lack of informed consent; lack of confidentiality and privacy, and neglect and abandonment.

##### Lack of informed consent

Several women reported that they were not given sufficient information regarding the progress of their labour and did not give their consent to certain procedures.

*“My first child…I had no strength to push the baby, so she told me she will inject me. She later cut me, then informed me I have cut you so that you are able to push, so I was informed after the cut but I think I should have been informed before” (FGD, Women).*

##### Lack of confidentiality and privacy

In addition to the issue of women’s HIV status being made known, communities perceive breaches of confidentiality as examination, delivery and treatment that required undressing without curtains or partitions.

*“There is no privacy because like in my case we shared a bed and when the doctor was coming to ask my problems there was no privacy the other patient would hear. Now like the problems of the one that we shared the bed with, I went home when I knew all her problems” (IDI, Woman).*

##### Neglect and abandonment

Abandonment was reported as the most common behavior that women and men felt was dehumanizing. Multiparous women reported that they are often left to deliver on their own as health providers say they have previous experience in delivering:

*“They have a habit of asking if it’s first or second born. If you say third born, they say that you are used to giving birth, they leave you alone, you push the baby alone only for them to come to cut the cord after birth and weigh the baby” (FGD, Women).*

Other examples of abandonment described by both women and men include situations in which health providers ignore clients for several hours despite calls for help; or women deliver alone on the admission room benches while health providers come in much later when she has already delivered or is screaming for help. At times abandonment of care was described when health providers fail to respond to clients’ needs especially when they are in pain post-operative.

*“… That was after the cervix had opened up and the baby had started coming out. So they were telling me to stop pushing and wait for the doctor yet the baby had started coming out. Like you may be feeling pain and you are calling out ‘doctor, doctor’ then they just say to each other ‘leave that one alone we are used to her’. So by the time they come the baby may have already come out” (IDI Woman).*

*“The provider will tell you sit down and she/he will continue with their own things while you are going through labor pains and when you are almost delivering you have to make noise to alert them and sometimes you may end up delivering the baby alone without knowing and they will quarrel you” (FGD, Women).*

When women are told they need a cesarean section they may have to wait for hours or for a whole night for the doctor on call to arrive and conduct the operation. Most men reported having to take their wives to another hospital because they felt they could not wait that long.

*“Women are neglected. They are told to wait for the doctor who is coming for the next shift and at that time one is in pain which cannot wait. So at that point you opt to go to another hospital” (FGD, Men).*

#### Poor rapport between women and health providers

Three main themes were drawn from the data regarding rapport and include lack of autonomy, detainment for non-payment of services following delivery, and ineffective communication.

##### Lack of autonomy

Common forms of lack of autonomy include lack of provision of food, drinks and bathing water at facilities*.* Sometimes, women have to queue in the ward to get food, and for those women who are in pain, and cannot move quickly enough, they miss out on meals. For example, women who have had a cesarean section reported that they were unlikely to get food and relied on friends and relatives to bring them food. The facilities lack water for bathing forcing relatives to carry water for bathing. Women felt that provision of food, hot drinks and warm water for bathing is important and failure to do so is disrespectful.

*“I was not given any hot drink to take after delivery, no water for bathing and my relatives brought me water for bathing” (FGD, Women).*

Women were reported to be required to undress in front of other women in wards, sharing beds and lack of assistance to carry baby soon after delivery.

“*We were sharing a bed, four people, so even we could not sleep. You just place the baby on the bed then you sit down. Since the bed is so small and already you are four so you just sit” (IDI Woman).*

##### Detainment

Our findings show that detainment due to inability to pay for services, though illegal, was a common practice. *“I found women who had been detained; one of them had been detained for over a month and had twins” (FGD Men).* While they are detained some women report that they are forced to work in the facilities (washing utensils, toilets and washroom). Others said they are not provided with beds and only the infant is given accommodation. They are separated from their infants and only allowed to breastfeed the baby at fixed times. Most of the detained mothers are not given food. This specific fear of detention was reported to influence women’s decision to deliver at home or with a traditional birth attendant. “*Lack of money also makes some mothers avoid going to hospital” (FGD Women).*

In some facilities, women are not detained but the facilities keep their identification cards. These women reported that they are then not able to seek further medical care or other social services since they lack their identification cards.^2^ Although some women found ways to avoid payment.

*“You see, you have no finance, so what you do, you put the baby in a hand bag and walk out of the hospital without their knowledge” (FGD Women).*

##### Ineffective communication

One critical theme that emerged is that women felt disenfranchised due to insufficient information sharing by the health providers resulting in poor understanding of procedures of childbirth leading to a feeling of helplessness and not in control of their bodies.

*“…and then there are other people who understand things slowly… the other time she will talk harshly… or will be harsh towards you maybe she sees you are not doing what she has told you, and maybe you have not understood. So, when you are in that pain they may tell you something and you do not hear…then she begins being harsh” (IDI Woman).*

First time mothers are not comfortable with positions of delivery and do not understand the need for frequent vaginal examinations (the standard is every four hours to assess descent of the baby’s head) and especially when they are not asked permission. Most health providers did not agree that they detain clients, but argued women are abandoned in hospitals by their relatives as some expressed;

*“Detention happens not only in XX but other areas, maybe the mother has come she delivered well and not paid anything. She will depend on the relatives and those people who are supposed to pay for her to look for money. Sometimes you don’t see the relatives and you find that no one is prepared to take the client home” (IDI, Service provider).*

*“When clients are unable to foot hospital bills, at times the clients are abandoned by their relatives in hospital. They are retained in the facility not because of the money” (IDI Service provider).*

#### Health system conditions and constraints

##### Lack of resources

Findings from FGDs show that, most public facilities were very dirty and they (women) are exposed to very unhygienic conditions including lack of water, poor sanitation and dirty labor and delivery rooms. Reports also include dirty water flowing in the wards:

*“The beds are too dirty …you see this room with curtains, it’s full of blood though they wipe the outside part; I saw them wipe the outside parts. If you see the bed itself you won’t enter inside it’s so dirty, they do remove the blanket and then wipe but it’s dirty.*

*Beds, even the washrooms are very dirty…the toilets have blocked and you can't even go inside.*

*Even from the entrance door it is full of water, washrooms are also flooded and then they come chuck you out in the morning” (IDI, Woman).*

##### Facility culture

The group dynamics of the maternal health team contribute to the culture of the facility – and is often influenced by both more senior or experienced health providers and the length of time they have spent in the facilities. Weak management and lack of accountability regarding a clean environment, sufficient equipment, commodities and drugs for health providers to do their work effectively; a poorly effected duty roster that does not maximize staffing ratios leading to a high workload; and ineffective supervision to ensure adherence to standards of care are some of the identified drivers of D&A that are tied up in the context of each facility culture.

##### Corruption

In some facilities ‘under the table payments’ are a common day to day practice with a hierarchy as to who receives what at the end of the day. Bribery was perceived as disrespectful for those who lack cash to bribe, but others (especially men) felt that it helped them acquire the services for their spouse including faster treatment and specialized care and privileges (such as not sharing a bed).

*“You give the nurse about 200 Kenya shillings so that she can treat you well. For me I took my wife to hospital with a motorbike and since I knew it will not take long I gave the nurse 200 shillings so that she can attend to her well. You must stretch your hand for quick processing”(FGD, Men).*

### Drivers of disrespect and abuse during childbirth

The qualitative findings also demonstrated several drivers for D&A as perceived by the local communities, health providers and managers. The driver themes drawn from the data are clustered around five main categories that build from community, health system, and policy levels. Community level includes individual, family, and community drivers. The health system level combines governance and leadership, management, service delivery and health provider drivers. The policy level includes drivers derived from national laws, policies, human rights and ethics.

### Individual, family and community level

#### Individual level drivers

Many of the women interviewed did not know their rights and what kind of treatment to expect from health providers. The poor are easily identified by their mode of dress and their inability to pay and are often discriminated or abandoned when seeking delivery services. The provider-client power dynamics also describe the context of expectations of social norms. For example, some women reported they were unable to correct or complain to a health provider about poor treatment.

*“You know why we say it is normal, when you go to a place, you follow the authority above, good or bad, you follow it” (FGD, Women).*

#### Family level drivers

Abuses at home perpetuated by social norms and gender inequalities contribute to the normalization of D&A experienced at facility during delivery. Data shows that the inability of women to defend rightful treatment coupled with poor understanding of patients’ rights, inadequate and complex legal redress mechanisms limits women and their families from asking or seeking retribution. It was strongly felt by all respondents that lower socio-economic status of the client was one of the main drivers for discrimination.

#### Community level drivers

Social norms such as gender inequalities also tend to disempower women creating skewed relations that may influence practices and expectations beyond the household and community and are often transferred to facility level when seeking care.

*“Even those insults that you hear they are not to harm the woman, but you do not do it out of fear or lack of knowledge you just allow the provider to do what they want. If they want to walk around the hospital let them walk when they feel like attending to her they will” (FGD, Men).*

The interaction between health providers and clients illuminated several insights that are illustrative of broader social cultural norms mentioned earlier. Majority felt that questioning a provider may subject them or their families to poor health services.

*“In hospital if you do not bribe the doctor and you have a very sick patient who needs quick assistance, you have to hold one of the nurses and request them to assist your patient. So if you want your patient to be treated well you have to keep quiet, submit and do as you are told” (FGD, Men).*

This lack of autonomy and disempowerment of community members discourages anyone from taking action or alternative measures after experiencing D&A. The process of seeking legal redress is also seen as expensive and time demanding and many witnesses decline to participate for fear of backlash.

*“If we would get like two people the story can be pushed. People fear the courts they can’t stand in courts as witnesses, they fear. They feel they will spend a lot of time and they have children to care for. You may also be competing with someone with money. The person will just look at you and at the end he gives money and justice is not done or you are killed” (FGD, Women).*

*“It is because we do not know where to go or even start. If there was a place where when you are mishandled you can report (starting point) then it can be better but now we don’t we just know police station or court…and we have failed to go to the court because of lack of money” (FGD, Women).*

### Health system level drivers

There were several health system level challenges: governance and leadership, service delivery and health providers (including the culture of a particular health facility) that appear to contribute to D&A.

#### Governance and leadership

Poor management of the available human resources and or chronic understaffing was discussed as a contributor to D&A. Women who were interviewed through the case narratives and had experienced D&A reported that understaffing made health providers neglect them because other cases were perceived more urgent. Women appear to understand the reasons health providers do this – due to shortage of staff and limited infrastructure of the facilities. Some health providers complained of being overworked and having to cover many service areas including running ANC clinics as well as conducting multiple deliveries. This leads to demotivation of health providers that can be expressed in the form of disrespect transferred to their clients.

Poor supervision of staff was also associated with mistreatment. For example women reported that health providers skip night duties (either they don’t turn up or go for long breaks) because of lack of adequate supervision resulting in women feeling abandoned. Some participants complained of poor supervision of interns resulting in medical malpractice and ‘excessive’ vaginal examinations.

“*Just see… She just delivered while I saw her…I was not in much pain …they took the child and weighed it… but did not wipe her blood stains the way they usually do… I just saw them leaving her there… then she rose up and wiped herself…she wiped the bed. So I was wondering why they were doing that for her” (IDI Woman).*

Poor supervision of facilities also leads to poor forecasting of supplies and commodities, staff attendance and quality assurance. Majority of women reported that the physical conditions of facilities are a good example of how health system constraints affect use. Some women noted that during medical consultation and treatment the facilities do not have private rooms and lack curtains or doors. Poor supply of drugs and supplies, and commodity management as well as the lack of equipment. Having to share a bed and to undress in public due to the lack of curtains to ensure privacy was reported to contribute to D&A.

Policy makers acknowledged that although communities expect good treatment during childbirth, “*if the provider does not have curtains or blankets they can do nothing to ensure privacy”* while others lamented the bad state of facilities where care can be compromised.

“*How do you how expect a midwife to be in a good mood if she works with no breaks, many client to attend to in a dirty working environment” (IDI, Policy maker).*

#### Service delivery and health providers

The asymmetrical relationship during provider-client interactions reflect a phenomenon where health providers are rated as more highly educated and with ‘higher authority’ and therefore cannot be questioned. Majority of the men felt that health providers are aware of the laxity of the Kenyan courts to take legal action on health providers and that the health providers use this to silence clients. Further they reported that abusive health providers are protected by the existing medical board and nursing and clinical officers’ councils who protect their colleagues and therefore no action is taken.

*“You know when you see them that thing is still in your heart, there is nothing you can do to them. Like I was asked by one provider ‘what court have you ever seen a doctor been taken’. When you look at it you see it is true because maybe the rich can know where to get lawyers that deal with such issues, but the poor will not know and also they do not have the money for that. So you just hold a grudge against them but there is nothing you can do” (FGD, Men).*

### National laws and policy level drivers

Our findings demonstrated that poor leadership and stewardship contribute to the weak implementation of existing policies and apparently very little accountability. Policy makers reported corruption within the system but there was apparently no formal redress for poor care contributed to mistreatment.

“*The managers are corrupt and the health providers know this. The health providers get nothing from the system other than the poor pay while at the level of county they use the little available resources meant to improve the working conditions” (IDI, Policy maker).*

*“Managers lack leadership qualities in dealing with D&A. They are also not accountable themselves so they lack moral authority to discipline any one. The government is not doing enough to deal with D&A” (IDI, Policy maker).*

Weak or non-existent legal redress mechanisms to deal with D&A continues to perpetuate the problem. Failure to uphold professionalism (i.e. adherence to standards) resulting from ignorance of ethical and human rights issues, and the perceived inadequacies of training institutions to either promote a good ‘bedside manner’ or inculcate professionalism appears to lead to a ‘don’t care attitude’ among health providers.

## Discussion

These findings build on the rapidly expanding literature-base on the extent of mistreatment of women during facility based childbirth globally. We used the framework developed by Bohren et al to tease out the most frequent observed/reported forms of D&A that emerged from qualitative interviews with communities and health providers in 13 facilities across five counties in Kenya (see Table 2). The primary forms of D&A reported in this study were undignified language (verbal abuse), physical abuse, abandonment and detention. These are similar to findings in Tanzania and Ghana [14, 15] and South Africa where negative client – provider interactions, inattentive staff and humiliation are described [9]. Although our data illustrates a good fit with the typology by Bohren et al [2], it also illuminates the complexity of understanding and the differences of how women, men and health providers describe the occurrence of mistreatment of women during childbirth in the Kenya setting.

There is concurrence of what may constitute each type of D&A as described by the third order themes: physical, verbal (and sexual) abuse, stigma and discrimination, failure to meet professional standards of care, poor rapport between women and providers, and health system constraints. However, the manifestations of experiences and reports (first order themes) vary depending on who reports or how it is reported. For example, some women and providers agree on the ‘need’ for slapping to save the babies life, while other women describe it as abuse - but the act of slapping itself appears to be normalized. Moreover, there was general agreement by community members, providers and their managers, that weak infrastructure and lack of equipment were contributors to mistreatment. Drivers of D&A at facilities range from individual provider to organizational and systems factors. These examples illustrate the need to contextualize the typology in order to develop strategies to reduce D&A at all levels [2, 11].

These insights have implications for developing strategies to promote respectful maternity care [16]. For example: women describe how health providers blame them for negative outcomes (of the pregnancy), and reprimand them for calling for help indicating the manifestations of power relations between some health providers and women in maternity settings that are of hegemonic dominance, and parallels the societal position of dominance of men [17–19]. Bohren et al also noted that several studies reported various nuances of verbal abuse that may have implications on the broader societal norms and expectations [1, 12]. This according to some authors is a reflection of broader features of violence against women that stems from structural gender inequality that systematically devalues the lives of women and girls [17]. Similarly, verbal abuse especially when linked to accusatory comments made to women about their sexual activity, operates in two main third order themes; verbal abuse and discrimination by age. Our data shows that such experiences were common to women who give birth when young and without partners or older women who are perceived to be ‘too old’ and should stop giving birth – i.e. discrimination based on parity.

The interrelationship of the first order themes presents challenges of developing specific strategies to reduce D&A. It also means that such unnecessary humiliations engender feelings of shame and is not conducive to establishing trusting connections with caregivers [12, 17]. Women are tired of being insulted and slapped and therefore will be reluctant to use facility based care in the future.

The need for contextualizing different experiences and reports from men, women and health providers is also illustrated by the additional categories of abandonment described when health providers fail to respond to women’s needs especially when they are about to give birth or when they are in pain post-operative. Both men and women described how student nurses lack the skills to attend to women in labour but also need to ‘practice’ on the same women. Moreover, it is apparent that many participants felt the skills of health providers were also lacking. This coupled with the fact that women reported loss of autonomy when denied access to medical information about the birthing process. This disempowered women and reduced them to states of passivity in which they were unable to be active participants in their own birth experience [17]. This loss of autonomy is often extended beyond the facility when in some facilities, women are not detained (because they cannot pay the fees) but the facilities keep their identification cards until they pay the full amount. These women are unable to seek further medical care or other social services since they lack their identification cards. This extension is a gross violation of the human rights.

Our data indicates that understanding the immediate triggers of D&A and the link with the drivers and consequences are critical pointers to developing interventions. Immediate triggers like ‘desire to save lives’ tend to develop ‘normalized’ behaviors that become behavior patterns that are difficult to understand among and between clients and health providers [14]. Another example of a trigger is the location where the mother lives or her dress code that may lead to discrimination as a result of perceived socio-economic status. Age of the women, marital status and women’s parity may trigger some form of discrimination that is linked to broader societal norms and expectations. Understanding the triggers are important while designing interventions as they point to potential behaviors that are either likely to be difficult to eradicate or easier to dealt with. The consequences of these actions (described in Table 3) are also varied from deterring future utilization of facility deliveries to by-passing nearby facilities through self-referrals that may pre-dispose women to more dangers.

**Table 3:** Linkages between the third order themes, triggers, drivers and consequences

Third order themes	Immediate triggers	Underlying drivers	Consequences of event
Physical abuse	Desire to save lives	Normalization Frustration of health providers associated with broader health system constraint/environment Gender inequalities and inadequate understanding of individual rights	Deter future facility delivery
Verbal abuse	Negative patient outcomes triggering some form of reprimand Desire to save lives	Normalization Complex legal redress mechanisms Frustration of health providers due to health system constraints- weak governance structures and accountability	Deter future facility delivery
Stigma and discrimination	Multiparous women Geographical location of women- poor neighborhoods/dress code Age –young teenagers Surnames HIV status	Broader community and societal/social economic challenges Tribalism Lack of knowledge or of patients’ rights	By passing nearest facilities leading to potential morbidities and mortalities associated with delays during self-referrals Deter future facility use
Failure to meet professional standards of care	Multiparous women Geographical location of women- poor neighborhoods/dress code Age –young teenagers	Health system drivers- Human resource challenges, weak legal and policy implementation Broader social cultural norms	By passing near facilities leading to potential morbidities and mortalities associated with delays during self-referrals Deter future facility use
Poor rapport between women and health providers	Geographical location of women-poor neighborhoods/dress code	Health system drivers- Human resource challenges Tribalism	By passing nearest facilities leading to morbidities and mortality associated with delays during self-referrals Deter future facility use

Following collection of this data (including quantitative data on prevalence of D&A) [13, 20] intervention development occurred through a participatory process that included community dialogues and stakeholder meetings to discuss the findings and engage all stakeholders in developing the interventions at community, health system and policy level. There was not one ‘silver bullet’ intervention to address all drivers – more a multi component intervention based on a human rights approach to address the multiplicity of causes of D&A. This multi component intervention is described in detail elsewhere [16, 21, 22], but includes strengthening quality improvement teams, values clarification and attitudes transformation workshops for managers and health providers to critically self-evaluate and modify their values, attitudes and beliefs; stress management counseling for providers; awareness raising around rights and obligations at community level and strengthening linkages between facilities and communities through maternity open days [22].

Developing interventions to prevent occurrence of D&A need to be built on a solid understanding of the context of the experiences and the root causes that should be explored using multiple methods including qualitative methods (such as community dialogue) throughout the intervention period. The interventions should focus on both*: intentional mistreatment* such as use of violence: physical, verbal, negligent withholding of care; and *structural disrespect*: deviations from accepted standards for infrastructure, staffing, equipment and supplies as well as unnecessary interventions demand for bribes and detention as described by Jewkes and Penn-Kekana [17].

Accountability at both policy level and management level are critical. There are very limited opportunities for those who have experienced D&A to pursue legal redress. Both men and women described the costs, fear and other barriers to challenge the health system through the courts. One policymaker also indicated that the frontline health providers were also treated badly by the system. Majority of health providers are members of professional associations – but seem not to adhere to the national or even international guidelines and ethics. For example the International Confederation of Midwives states: “*This code acknowledges women as persons with human rights, seeks justice for all people and equity in access to health care, and is based on mutual relationships of respect and trust, and the dignity of all members of society”* [23].

Use of behaviors interventions to reduce drivers of *intentional mistreatment* has shown positive results in Kenya [21]. Busy maternity units are stressful environments – particularly so when demand exceeds supply; frequently there are insufficient health providers available which leads to perceptions of abandonment and neglect. One nurse/midwife can only deliver one baby at a time. Providing opportunities for health providers to receive counseling in groups or individuals has made a difference in helping them cope in difficult situations [16, 24]. Moreover raising awareness among communities and updating providers on both health obligations and rights can improve communication between women and health providers [22]. However additional efforts need to be made to address the drivers of s*tructural disrespect*. This requires substantial investment to improve structural features such as the absence of curtains or screens that contribute to lack of confidentiality or privacy. Equipment shortages – especially lack of sufficient beds that contribute to limited confidentiality and privacy – as women sharing a bed overheard each other’s interaction with the health providers.

The use of Bohren typology to elucidate the drivers of D&A is relevant and provides a good starting point to develop strategies to reduce occurrence of D&A [2]. Drivers described at five different levels (individual, family, community, health system and policy levels) have complex interactions with the societal influence and context. Power imbalances and gender disparities also have a role to play; until women are empowered and have agency to both demand quality maternal health care and respect, addressing the structural deviations and intentional mistreatment will have limited influence on improving socio-health issues for pregnant women.

Using the third order themes is likely to provide standardized estimates across different settings. However, the manifestation of first order themes are critical to contextualize as they are closely linked to triggers and drivers that are relevant for developing interventions.

There are a few limitations to this study. We do not include results of interviews with women who did not report D&A so did not fully unpack the drivers of respect. All occurrences of mistreatment are self-reported which could be either over or under-reported. However, during the development of the interventions in the broader project we discussed these qualitative findings through community dialogues in each location to verify the reports. One other limitation is using the Bohren framework retrospectively. Although given the challenges around this emerging issue on ways to measure prevalence, developing definitions, and interventions, we feel it is a pretty good fit.

## Conclusion

The results described here build on the ever-expanding literature on mistreatment of women during labour and childbirth –outlining drivers from an individual, family, community, facility and policy level. New frameworks to group the manifestations into themes or components makes it increasingly more focused on specific interventions to promote respectful maternity care. The Kenya findings resonate with budding literature – demonstrating that this is indeed a global issue that needs a global solution.

## Abbreviations

ANC: Antenatal care
D&A: Disrespect and abuse
FGD: Focus group discussion
IDI: In depth interview
TBA: Traditional birth attendant

## Footnotes

1 In 2013, a Presidential decree, mandated free maternity services in Kenya.

2 Since 2013 maternity services are now free in Kenya – meaning no one can be detained for lack of payment.

## References

[1] Kujawski S, Mbaruku G, Freedman LP, Ramsey K, Moyo W, Kruk ME (2015). Association between disrespect and abuse during childbirth and Women's confidence in health facilities in Tanzania. Matern Child Health J.

[2] Bohren M, Vogel JP, Hunter EC, Lutsiv O, Makh SK, Souza JP, Aguiar C, Saraiva CF, Diniz AL, Tuncalp O, Javadi D, Oladapo OT, Khosla R, Hindin MJ, Gulmezoglu AM (2015). The mistreatment of women during childbirth in health facilities globally: a mixed-methods systematic review. PLoS Med.

[3] Bowser D. & Hill K. Exploring evidence for disrespect and abuse in facilitybased childbirth: report of a landscape analysis. USAID-TRAction Project, Harvard School of Public Health and University Research Co., September 20, 2010. Available at http://www.traction.org. 2010.

[4] Asefa A, Bekele D (2015). Status of respectful and non-abusive care during facility-based childbirth in a hospital and health centers in Addis Ababa. Ethiopia Reprod Health.

[5] Abuya T, Warren CE, Miller N, Njuki R, Ndwiga C (2015). Exploring the prevalence of disrespect and abuse during childbirth in Kenya. PLoS One.

[6] Okafor II, Ugwu EO, Obi SN (2015). Disrespect and abuse during facility-based childbirth in a low-income country. Int J Gynaecol Obstet.

[7] Misago C, Kendall C, Freitas P, Haneda K, Silveira D, Onuki D, Mori T, Sadamori T, Umenai T (2001). From 'culture of dehumanization of childbirth' to 'childbirth as a transformative experience': changes in five municipalities in north-east Brazil. Int J Gynaecol Obstet.

[8] Lukasse M, Laanpere M, Karro H, Kristjansdottir H, Schroll AM, Van Parys AS, Wangel AM, Schei B (2015). Bidens study, group, *pregnancy intendedness and the association with physical, sexual and emotional abuse - a European multi-country cross-sectional study*. BMC Pregnancy Childbirth.

[9] Silal S, Penn-Kekana L, Barnighausen T, Schneider H, Harris B, Birch S, McIntyre D (2012). Exploring inequalities in access to and use of maternal health services in South Africa. BMC Health Serv Res.

[10] Sando D, Ratcliffe H, McDonald K, Spiegelman D, Lyatuu G, Mwanyika-Sando M, Emil F, Wegner MN, Chalamilla G, Langer A (2016). The prevalence of disrespect and abuse during facility-based childbirth in urban Tanzania. BMC Pregnancy Childbirth.

[11] Freedman LP, Kruk ME (2014). Disrespect and abuse of women in childbirth: challenging the global quality and accountability agendas. Lancet.

[12] Freedman L, Ramsey K, Abuya T, Bellows B, Ndwiga C, Warren CE, Kujawski S, Moyo W, Kruk ME, Mbaruku G (2014). Defining disrespect and abuse of women in childbirth: a research, policy and rights agenda. Bull World Health Organ.

[13] Warren C, Rebecca Njuki, Timothy Abuya, Charity Ndwiga, Grace Maingi, Jane Serwanga, Faith Mbehero, Louisa Muteti, Anne Njeru, Joseph Karanja,, Joyce Olenja, Lucy Gitonga, Chris Rakuom, and Ben Bellows. Study protocol for promoting respectful maternity care initiative to assess, measure and design interventions to reduce disrespect and abuse during childbirth in Kenya. BMC Preg and Childbirth. 2013;13:21. doi: 10.1186/1471-2393-13-2. 10.1186/1471-2393-13-21 PMC3559298 23347548

[14] McMahon S, George AS, Chebet JJ, Mosha IH, Mpembeni RN (2014). Experiences of and responses to disrespectful maternity care and abuse during childbirth; a qualitative study with women and men in Morogoro Region, Tanzania. BMC Pregnancy Childbirth.

[15] Moyer CA, Adongo PB, Aborigo RA, Hodgson A, Engmann CM (2014). They treat you like you are not a human being': maltreatment during labour and delivery in rural northern Ghana. Midwifery.

[16] Warren CE, Charity Ndwiga, Pooja Sripad, Melissa Meddich, Anne Njeru, Alice Maranga, George Odhiambo, and Timothy Abuya. Sowing the seeds of transformative practice to actualize women’s rights to respectful maternity care: reflections from Kenya using the consolidated framework for implementation research. BMC Womens Health - under review 2016. 10.1186/s12905-017-0425-8 PMC5577789 28854925

[17] Jewkes R, Penn-Kekana L (2015). Mistreatment of women in childbirth: time for action on this important dimension of violence against women. PLoS Med.

[18] Nasong'o S, Ayot TO. Women in Kenya’s Politics of Transition and Democratisation, in The Struggle for Democracy. S.W.N.o.E. G. Murunga, Editor. 2007, CODESRIA: Dakar, Senegal. p. 164-96.

[19] Muckle W, Sprague A, Muckle W, Sprague A, Fergus S (2013). Barriers to access of maternity care in Kenya: a social perspective. J Obstet Gynaecol Can.

[20] Abuya T, Maina T, Chuma J (2015). Historical account of the national health insurance formulation in Kenya: experiences from the past decade. BMC Health Serv Res.

[21] Abuya T, Ndwiga C, Ritter J, Kanya L, Bellows B, Binkin N, Warren CE (2015). The effect of a multi-component intervention on disrespect and abuse during childbirth in Kenya. BMC Pregnancy Childbirth.

[22] Ndwiga C, Warren C, Abuya T, Kanya L, Maranga A, Ochieng C, WanjalaM, Chelang’at B, Njeru A, Gituto A, Odhiambo G, Mbehero F, Maina L, and Jeremiah Maina. Respectful Maternity Care Resource Package: Facilitator’s Guide. 2015. Population Council New York.

[23] ICM. International Code of Ethics for Midwives. 2008 [cited 2016; Available from: http://www.internationalmidwives.org.

[24] Ndwiga C, WC, Ritter J, Sripad P, Abuya T. Exploring provider perspectives on respectful maternity care in Kenya: “Work with what you have”. Reproductive Health. 2017 under review. 10.1186/s12978-017-0364-8 PMC5567891 28830492

